# Evidence of perceived psychosocial stress as a risk factor for stroke in adults: a meta-analysis

**DOI:** 10.1186/s12883-015-0456-4

**Published:** 2015-11-12

**Authors:** Joanne Booth, Lesley Connelly, Maggie Lawrence, Campbell Chalmers, Sara Joice, Clarissa Becker, Nadine Dougall

**Affiliations:** Institute for Applied Health Research, School of Health & Life Sciences, Glasgow Caledonian University, Glasgow, Glasgow Caledonian University, Cowcaddens Road, Glasgow, G4 0BA UK; NHS Lanarkshire, Coathill Hospital, Hospital Street, Coatbridge, ML5 4DN UK; School of Psychology, Massey University, Private Bag 11 222, Palmerston North, New Zealand; Nursing, Midwifery & Allied Health Professions Research Unit, University of Stirling Unit 13 Scion House, Stirling University Innovation Park, Stirling, FK9 4NF UK

**Keywords:** Stress, Psychological, Stroke, Risk factor

## Abstract

**Background:**

Several studies suggest that perceived psychosocial stress is associated with increased risk of stroke; however results are inconsistent with regard to definitions and measurement of perceived stress, features of individual study design, study conduct and conclusions drawn and no meta-analysis has yet been published. We performed a systematic review and meta-analysis of studies assessing association between perceived psychosocial stress and risk of stroke in adults.The results of the meta-analysis are presented.

**Methods:**

Systematic searches of MEDLINE, EMBASE, CINAHL, PsycInfo, and Cochrane Database of Systematic Reviews were undertaken between 1980 and June 2014. Data extraction and quality appraisal was performed by two independent reviewers. Hazard ratios (HR) and odds ratios (OR) were pooled where appropriate.

**Results:**

14 studies were included in the meta-analysis, 10 prospective cohort, 4 case–control design. Overall pooled adjusted effect estimate for risk of total stroke in subjects exposed to general or work stress or to stressful life events was 1.33 (95 % confidence interval [CI], 1.17, 1.50; *P* < 0.00001). Sub-group analyses showed perceived psychosocial stress to be associated with increased risk of fatal stroke (HR 1.45 95 % CI, 1.19,1.78; *P* = 0.0002), total ischaemic stroke (HR 1.40 95 % CI, 1.00,1.97; *P* = 0.05) and total haemorrhagic stroke (HR 1.73 95 % CI, 1.33,2.25; *P* > 0.0001).A sex difference was noted with higher stroke risk identified for women (HR 1.90 95 % CI, 1.4, 2.56: *P* < 0.0001) compared to men (HR 1.24 95 % CI, 1.12, 1.36; *P* < 0.0001).

**Conclusions:**

Current evidence indicates that perceived psychosocial stress is independently associated with increased risk of stroke.

## Background

There is a lack of attention paid to the potential role of psychosocial risk factors, including perceived psychosocial stress, in the development of stroke [1, 2]. The association between psychosocial stress and the development of coronary heart disease is strong [3–5]. A recently published overview of systematic reviews confirms modest to moderate evidence of the association between psychosocial stress at work and cardiovascular outcomes [6]. Research is less conclusive in the area of stroke, yet public perception highlights psychosocial stress as a key risk factor for stroke [7–9]. Several observational studies have identified an association between perceived psychosocial stress and stroke, [10–13] however conflicting findings have been reported and inconsistencies are apparent with respect to definition and measurement of perceived psychosocial stress, study design and quality, duration of follow-up and number of covariates adjusted for. This variability has resulted in different, and sometimes contradictory, conclusions being drawn about the relationship between perceived psychosocial stress and stroke [14–16]. There is no clarity of clinical message about any possible contribution of perceived psychosocial stress to stroke risk and a lack of evidence around the potential for stress modification interventions for primary or secondary prevention of stroke [17]. To date, no systematic review or meta-analysis of studies reporting associations between perceived psychosocial stress and stroke has been published. Psychosocial stressors, caused by relationship, occupational or financial-related stimuli, are recognised as potential contributors to an individual’s perceptions of stress, which is the human response to exposure to psychosocial stressors and inability to cope with the demands made [18, 19]. However psychosocial stressors are under-investigated compared to more established biological and pathophysiological risk factors for stroke [17] and the complexity of the relationship between exposure to psychosocial stressors and perception of psychosocial stress is not fully understood. Studies have reported various sub-components of psychosocial stress, including self-perceived stress [12, 14–16] stressful life events (SLE) [11, 13] and poor adaptation to stress [10] to be associated with an increased risk of stroke. We performed a systematic review and meta-analysis to evaluate the association between perceived psychosocial stress and stroke, and to clarify differential risks associated with types of stroke and sub-components of perceived stress. Stress as a trigger for stroke events was not included as this was not considered a component of risk. This paper reports the results of the meta-analysis.

## Methods

The meta-analysis was undertaken according to the proposal for reporting Meta-analyses Of Observational Studies in Epidemiology (MOOSE) [20].

### Search strategy

**Fig. 1:**
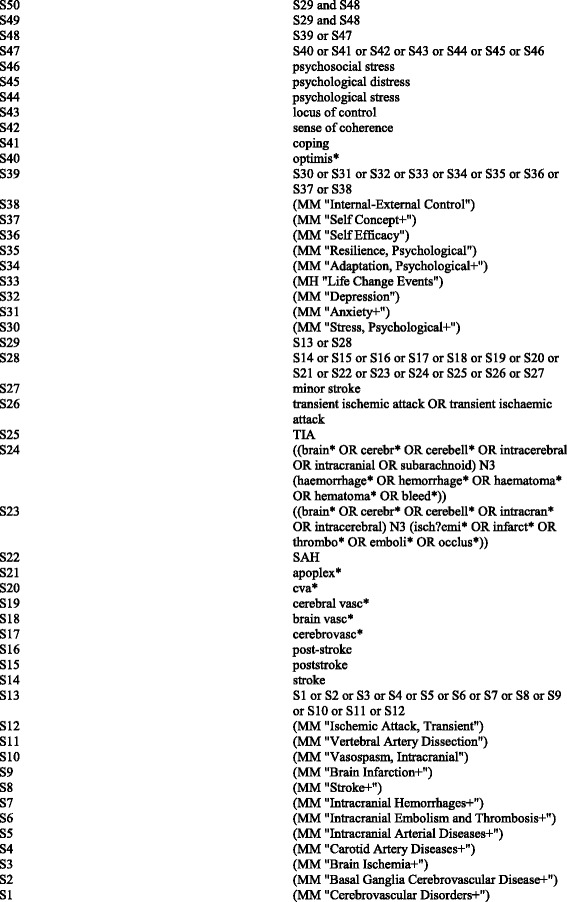
Search strategy: MEDLINE (EBSCO), June2014

Systematic searches of published papers indexed in MEDLINE, EMBASE, CINAHL, PsycInfo and Cochrane Database of Systematic Reviews between 1980 and June 2014 were undertaken using a strategy combining selected subject headings and keywords relating to perceived psychosocial stress and stroke. The search strategy was developed for use in Medline (Fig. 1) and amended for use in other databases. Manual searching of reference lists and relevant systematic reviews and guidelines, was also performed. Results were filtered for English language.

### Study selection

Studies were included in the meta-analysis if they met the following criteria: (1) prospective cohort or case–control design (2) self-reported exposure to SLE or self-reported perceived psychosocial stress or self-reported work stress or self-reported exposure to high strain work (3) use of adjusted models or matching procedures that controlled for potential confounders (4) reported risk estimates for stroke outcomes with 95 % CI comparing participants who had experienced perceived psychosocial stress to participants who had not experienced perceived psychosocial stress or who had experienced perceived psychosocial stress to a lesser degree. Studies reporting a clinical diagnosis of depression or clinical diagnosis of anxiety were excluded, as were studies involving the composite construct of psychological distress unless a measure of perceived psychosocial stress could be extracted. In the case of overlapping data within and between studies, the report with the longest follow-up or largest number of participants was included. Studies were included in the meta-analysis if they reported risk estimates adjusted for at least three stroke risk factors. A broad definition of stroke was adopted, to include ischaemic stroke, haemorrhagic stroke, subarachnoid haemorrhage and TIA [21]. The definition of perceived psychosocial stress used was ‘the self-reported sensation of tension, irritability, nervousness, anxiety or sleeplessness [12] associated with poor health, family relationships, living arrangements, finance, work and stressful life events’. Eligible studies were selected by a two stage process. Using the broad criteria of stroke and stress, two reviewers (from JB, LC, ML) independently screened titles and abstracts, where available, of bibliographic records retrieved. Full-text copies of potentially relevant studies were retrieved. Two reviewers then used the pre-determined selection criteria to assess eligibility. Disagreement was resolved by discussion with a third reviewer.

### Data extraction and quality appraisal

Two reviewers extracted data independently (from JB, LC, ML, CC, SJ, CB) using a review-specific extraction tool. Data to be extracted included details of study design and methods; study populations including sex and age; types of stress exposure and method of measurement; stroke outcomes and measurement or confirmation method; number and type of confounders adjusted for and sensitivity analyses. Extracted data were cross-checked and disagreements resolved by consensus. Where indicated, authors were contacted and asked to provide missing information. Independent assessment of methodological quality was conducted using the Newcastle-Ottawa Quality Assessment Scales for Cohort Studies and Case–control Studies [22] to grade selection of participants, assessment of exposures and outcomes, and comparability and control of confounding. The maximum total score is 9.

### Data synthesis

For studies which reported adjusted risk estimates, a meta-analysis was performed to pool estimates of association. For cohort studies, hazard ratios (HRs) were used as the common risk estimate across studies (relative risks were considered equivalent to HRs). For case–control studies, odds ratios (ORs) were used as the common risk estimate. If different adjusted risk estimates were reported, the most fully adjusted estimate was included. Forest plots were produced to visually assess the association across the included studies and the corresponding 95 % CI. The chi-squared test was employed to determine strength of evidence that heterogeneity was genuine, where *P* < .10 was considered indicative of statistically significant heterogeneity. The I^2^ statistic was used to quantify inconsistency, the percentage variability in effect estimates due to heterogeneity between studies rather than sampling error within studies. An I^2^ value over 50 % may indicate substantial heterogeneity. Pooled results were estimated using a random-effects model as this provides a more conservative estimate of exposure effect where there is a high likelihood of substantial between-study variance (DerSimonian and Laird model) [23]. Possibility of publication bias was evaluated by visual inspection for possible skewness in a funnel plot, and Egger regression [39] was used to judge the degree of publication bias. Sub-group analyses were undertaken for gender, type of stress exposure and type of stroke. Sensitivity analyses were performed to analyse influences of specific study characteristics. Analyses were performed using Review Manager Version 5.2 [24].

### Results

#### Literature search

**Fig. 2:**
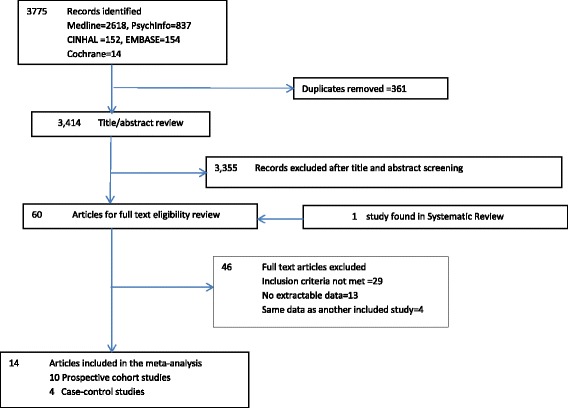
Study selection flow chart

The search strategy identified 3775 citations, of which 14 were included in the meta-analysis, 10 prospective cohort studies (145,546 participants, 5725 stroke outcomes) and 4 case–control studies (4405 stroke cases, 4987 controls) (Fig. 2). In total 10,130 stroke outcomes from 154,938 participants were included in the meta-analysis.

### Study characteristics

Characteristics of the studies are shown in Table 1 (cohort) [2, 3, 14, 25–28, 30, 31, 33] and Table 2 (case control) [15, 16, 29, 32]. Two prospective cohort studies reported results separately by sex with no risk estimate reported for the combined male and female cohort [3, 25]. Of 8 studies reporting results for a total sample of men and women, 2 studies [15, 28] provided stratified results by sex. The studies were undertaken across the full adult age range with 8 cohort studies reporting results for adults aged 40 and over [2, 3, 26–28, 30, 31, 33], 1 for adults aged 18 and over [25] and 1 for those aged 20 and over [14]. Three of the 4 case control studies included adults aged 18 and over [15, 16, 29] and the eligible age for the fourth was 39. Characteristics of the stress exposure are shown in Tables 1 and 2. Thirteen studies measured stress exposures through self-report questionnaires, 1 study used a structured interview schedule [2]. The majority used study specific questionnaires but provided no information regarding validity or reliability. Five studies used validated tools [25, 26, 29, 31, 32]. Two studies looking at general stress used a single item questionnaire however no information was reported regarding its validity or reliability as a means of measuring stress [15, 16].

**Table 1:** Characteristics of included cohort studies

Author YearCountry	Quality rating	Cohort size (n)Baseline age range (y)	% Male	Stroke cases (*n*)Follow-up years (y)	Stress exposure and measure	Stroke outcomes	Baseline stroke excluded	Number of confounders controlled for in adjusted model
						Risk estimates Hazard Ratio (95 % CI)		
Harmsen, 2006 [33] Sweden	8	7457	100	1019	Self-perceived stress General stress	Fatal and non-fatal stroke	Yes	11
		47–55		28.0	Single question	1.25(1.03-1.52)		
Henderson, 2013 [31] USA	8	2326	38	414	Perceived stress General stress	Fatal and non-fatal stroke	Yes	10
		> = 65		6.0	6 item perceived stress scale [41]	1.08(0.97-1.20)		
Iso, 2002 [3] Japan, males	8	30,180	100	341	Perceived mental stress General stress	Fatal stroke	Yes	9
		40–79		7.9	Single question	1.12(0.78-1.61)		
Iso, 2002 [3] Japan, females	8	43,244	0	316	Perceived mental stress General stress	Fatal stroke	Yes	9
		40–79		7.9	Single question	2.24(1.52-3.30)		
Kornerup, 2010 [30] Denmark	8	9542	43	350	> 4 major life events in a life course	Fatal and non-fatal ischaemic stroke	Yes	11
		Mean M		6–9	SLE			
		56.6 (SD 15.5) F-59.1 (SD 15.4)			11 item self-report questionnaire	1.32(0.77–2.26)		
McLeod, 2001 [26] Scotland	7	5388	100	122	Perceived stress General stress	Fatal stroke	No	9
		35–64		21.0	4 item Reeder stress inventory [42]	0.98(0.55-1.75)		
Molshatzki, 2013 [2] Israel	7	10,059	100	665	Perceived hardships General stress	Fatal stroke	No	7
		> = 40		28.1	14 questions on work, family, finance	1.33(1.07-1.65)		
Ohlin, 2004 [28] Sweden	8	13,280	80	643	Permanent stress General stress	Fatal and non-fatal stroke	Yes	9
		Mean 45		21.3	2 questions	1.29(1.04-1.60)		
Suadicani, 2011 [27] Denmark	7	4943	100	779	Perceived psychological work pressure	Fatal and non-fatal stroke (excl SAH)	No	8
		40–59		30.0	Leisure stress	1.17(0.98-1.40)		
					3 questions			
Truelsen, 2003 [14] Denmark	8	12,574	45	929	Self-reported stress General stress	Fatal and non-fatal stroke	Yes	11
		20–98		14–16	2 questions	1.13(0.85-1.50)		
Tsutsumi, 2009 [25] Japan Males	8	3190	100	91	Occupational stress	Fatal & Non-fatal stroke	Yes	10
		18–65		11.0	11 item demand-control questionnaire	2.53(1.08-5.93)		
Tsutsumi, 2009 [25] females	8	3363	0	56	Occupational stress	Fatal & Non-fatal stroke	Yes	10
		18–65		11.0	11 item demand-control questionnaire	1.46(0.63-3.38)

**Table 2:** Characteristics of included case–control studies

Author YearCountry	Quality rating	Cases: controls Type of controls	% Male	Cases: controls with stress	Stress exposure and measure	Stroke outcomes	Previous stroke excluded	Number of confounders controlled for in fully adjusted model
						Risk estimates Odds Ratio (95 % CI)		
		Age range (y)						
Jood, 2009 [15] Sweden	7	600 : 600 Population 18–69	64	126:46	Permanent self-perceived psychological stress General stress	Non-fatal ischaemic Stroke	No	10
					Single question	3.49(2.06-5.91)		
O’Donnell 2010 [16] 22 countries	5	3000 : 3000	63	589:440	Psycho-social stress General stress	Non-fatal stroke (excludes SAH)	Yes	13
		Hospital or community			Single question	1.30(1.11-1.52)		
		Mean 61.1 (SD 12.7)						
Abel, 1999 [32] USA	5	655:1087	44.6 % cases, 39.9 % controls	Categorical boundary for stress risk factor not used so raw numbers ‘with stress RF’ not reported	20 point increase on GSRRS 35 item Geriatric Social Readjustment Rating Scale [43] General stress	Fatal & non-fatal ischaemic stroke	Yes	7
		Community						
		Mean age						
		Cases 69.8				1.01(0.99-1.03)		
		Controls 70.2						
Egido, 2012 [29] Spain	6	150:300	77.3 % cases, 36.3 % controls	62:50 (41.4 %:16.7 %)	Score > =150on Holmes & Rahe 40-item questionnaire of life events [41]	Non-fatal stroke (90 % were ischaemic)	Yes	9
		Population						
					8 item ERCTA (Recall Scale of Type A Behaviour [44]			
		Mean age						
		Cases 53.8						
						3.84(1.91-7.72)		
		(SD 9.3)						
		Controls 53.6						
		(SD 9.6)						

All studies confirmed stroke by death certificates, medical records, official registers or CT/MRI scan. Baseline stroke was not excluded in 4 studies [2, 15, 26, 27]; however a stratified analysis excluding these studies was undertaken*.* Stress exposure was measured once at baseline in 13 studies. McLeod et al. [26] performed a second screening 5 years post baseline. All risk estimates were adjusted for age, smoking status, BMI, and hypertension. The majority adjusted for physical activity [3, 14, 25–28, 30, 31, 33] (9 studies), diabetes [2, 3, 14, 25, 27, 30, 33] (7 studies), alcohol consumption [3, 14, 25–28] (6 studies) and cholesterol [25, 26, 28, 30, 31, 33] (6 studies). In addition to traditional biological and lifestyle factors associated with stroke, all except one study [3] adjusted for social factors such as educational attainment, occupational status or social class, whilst 2 studies [3, 30] adjusted for depression or psychological factors other than stress. All cohort studies were of high methodological quality, scoring 7 or above (of a maximum possible 9) on the Newcastle Ottawa Quality Assessment Scale. Case control studies were less robust, scoring 5–7 of the possible maximum of 9 on the Newcastle Ottawa Quality Assessment Scale.

### Stress as a risk factor for stroke

**Fig. 3:**
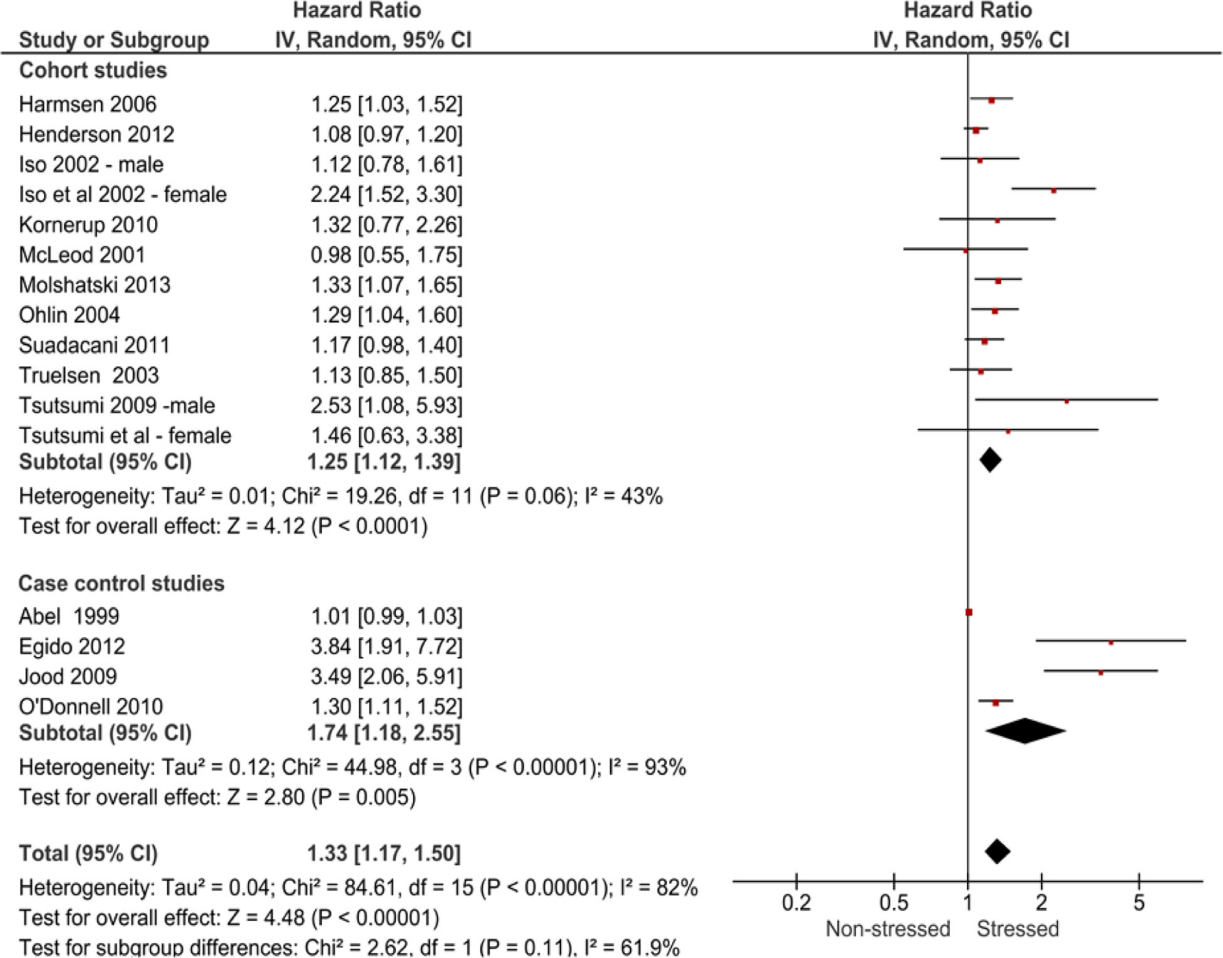
Forest plot of overall pooled adjusted effect estimate for risk of any type of stroke in subjects exposed to perceived stress

The overall pooled adjusted effect estimate for risk of total stroke in subjects exposed to general or work stress or to SLE versus control was 1.33 (95 % CI, 1.17, 1.50; *P* < 0.00001) with substantial statistical heterogeneity (I^2^ = 82 %; p value for Q test <0.00001). The pooled HR for the 10 prospective cohort studies was 1.25 (95 % CI 1.12, 1.39; *P* < 0.0001) with moderate statistical heterogeneity (I^2^ = 43 %; p value for Q test =0.06) and the pooled OR for the 4 case–control studies was 1.74 (95 % CI 1.18, 2.55; *P* = 0.005) with considerable statistical heterogeneity (I^2^ = 93 %; p value for Q test < 0.00001). Comparison between prospective cohort and case control studies revealed no significant difference (*P* = 0.11), indicating minimal methodological heterogeneity (Fig. 3).

### Subgroup analysis

Results by sex were available from 10 prospective cohort studies. For men, 6 of 7 reported associations were positive with a pooled HR of 1.24 (95 % CI, 1.12–1.36; *P* < 0.0001). Three studies reporting women only results showed positive associations and a pooled HR of 1.90 (95 % CI 1.40, 2.56; *P* < 0.0001). One case control study reported no difference between sexes [15]. The different types of stress were considered separately and for the purposes of analysis were categorised as general stress, work stress and stressful life events. In the 7 prospective cohort studies that reported it, the pooled HR for general stress was 1.25 (95 % CI, 1.09–1.42; *P* = 0.0009), for work stress 1.39 (95 % CI, 0.93, 2.10; *P* = 0.11), and for stressful life events 1.32 (95 % CI, 0.77, 2.26; *P* = 0.31). In the 2 case control studies that reported general stress the pooled OR was 2.06 (95 % CI, 0.78–5.41; *P* = 0.14), and for stressful life events it was 1.88 (95 % CI, 0.51–6.93; *P* = 0.34). No case control studies relating to work stress were eligible for inclusion in the meta-analysis.

When considering the outcomes, six cohort studies reported results for fatal stroke with a pooled HR of 1.45 (95 % CI, 1.19–1.78; *P* = 0.0002). Two studies reported results for non-fatal stroke with a pooled HR of 0.98 (95 % CI, 0.89–1.08). When the type of stroke was considered the pooled HR for fatal and non-fatal ischaemic stroke was 1.40 (95 % CI, 1.00–1.97; *P* = 0.05), and for fatal and non-fatal haemorrhagic stroke it was 1.73 (95 % CI, 1.33–2.25; *P* < 0.0001). In the case control studies the pooled OR for non-fatal ischaemic stroke in the 2 studies reporting results was 2.06 (95 % CI, 0.78–5.41; *P* = 0.14). Only one case control study reported on haemorrhagic stroke separately [15] with an OR of 1.23 (99 % CI, 0.89–1.69).

### Sensitivity analysis

Excluding the cohort study with the largest influence [3] and the cohort studies which did not exclude participants with baseline stroke [2, 26, 27] produced a similar pooled HR which remained statistically significant. Sensitivity analyses which excluded studies with less than 5000 participants [27, 31] or with less than 10 years follow-up [3, 30, 31] or those which did not control for diabetes [26, 28, 31] also produced similar pooled HRs, which remained statistically significant. Excluding the case control study with the largest influence [16] changed the result from statistically significant to non-significant (OR 2.30 95 % CI, 0.82–6.44; *P* = 0.11), as did excluding the case control study which did not exclude participants with baseline stroke [15] (OR 1.37 95 % CI, 0.98–1.92; *P* = 0.07). Analysis excluding the study with less than 500 cases [29] reduced the pooled OR to 1.46 (95 % CI, 1.01–2.10; *P* = 0.04) however it remained statistically significant.

### Publication bias

The funnel plot appeared asymmetric, a number of the smaller studies estimated larger hazard ratios than the larger studies, and there was evidence of possible publication bias using the Egger method (*p* = 0.000) [39].

## Discussion

Meta-analysis of 14 studies (10 cohort, 4 case–control) involving a total of 10,130 strokes found a positive association between perceived psychosocial stress and risk of stroke in adult men and women, suggesting that perceived psychosocial stress may be an independent risk factor for stroke. The combined pooled adjusted effect estimate showed a 33 % increased risk of incident stroke in those reporting perceptions of psychosocial stress and was statistically significant in the separate cohort and case–control study analyses. The increased risk is moderate, being of similar magnitude to risk associated with diabetes mellitus, dietary risk score or depression [16] when compared to the larger effect size associated with history of hypertension, current smoking, waist-to-hip ratio, alcohol intake, regular physical activity, cardiac causes and ratio of apolipoproteins B to A1. Psychosocial stress is an imprecise term which has multiple interpretations. There is no accepted, universal definition and, depending on perspective, subjective stress comprises physiological, emotional, motivational and cognitive elements all of which may indicate a degree of stress response [35]. In this review we chose not to narrowly define type of psychosocial stress. An inclusive approach was taken involving wide-ranging descriptions of perceived stress such as general stress, where type of stress was not detailed, occupational stress and major life events [34]. However the common essential element was the report of subjective stress, as perceived and self-reported by study respondents, not stress that was objectively assessed or measured by another means. The concept of individual perception of stress was important in the absence of any measure or biomarker for actual stress level.

Nevertheless the validity of the exposure measurements of perceived psychosocial stress can be questioned. The majority of the studies included in the meta-analysis used study-specific questionnaires and for many this comprised a single question. The focus of this was broad ranging covering such areas as perceived stress at home or work and including different intensities of exposure from some periods to permanent stress. It is questionable how sensitive the different measures of perceived stress were and indeed their discriminatory properties. A feature to be considered is the extent to which socioeconomic disadvantage was identified, both previous and current, because a sensitive measure of this may explain the observed increase in stroke risk when material disadvantage and associated behavioural hazards are taken into account.

Subgroup analysis to identify specific type of perceived stress associated with stroke was inconclusive with only general stress showing a clear association with increased risk. In nine of the ten cohort studies and two of the four case–control studies general stress indicated perceptions of chronic stress, rather than the result of an individual stressful event or accumulation of stressful life events. These findings potentially resolve some contradictions in the published evidence regarding the type of perceived stress associated with risk of stroke, demonstrating that ongoing perceived stress of a continuous or regular nature was associated with increased risk of stroke. The relative lack of studies measuring discrete life events in this meta-analysis leaves unanswered the question of whether discrete life events contribute to stress-associated stroke risk.

The eligibility criterion of requiring self-report of psychosocial stress limited the studies that could be included in the meta-analysis. This applied to studies of occupational stress in particular where workplace exposure to psychosocial stress was often determined according to a previously developed Job Exposure Matrix of demand and control for a diverse range of occupations rather than self-reported answers to questions about perceived psychosocial stress [45]. Thus the risks associated with perceived work stress remain speculative.

Subgroup analysis also confirmed that perceived psychosocial stress was linked to stroke in both sexes however higher risk was associated with female sex. There is no clear explanation for this difference and it is not known whether this result indicates that women are exposed to higher levels of stress, whether female perceptions of psychosocial stress are different to male, or whether their reporting experiences of stress are different. Our results concur with those from a literature review of work-stress related stroke among working women, which suggested that work stress may be a more powerful predictor of stroke among women than men [46]. However female sex specific data are limited and these findings should be investigated further to identify potential explanatory mechanisms. With regard to type of stroke, analysis revealed a significant association between perceived psychosocial stress and fatal stroke of all types but this relationship was not identified in the non-fatal stroke data. Both ischaemic and haemorrhagic stroke were associated with perceived psychosocial stress; however the stronger association was with haemorrhagic stroke. These results contrast with a large individual-participant data meta-analysis of occupational job strain and risk of stroke which found work stress to be associated with a 20 % increased risk of ischaemic stroke but no association with haemorrhagic stroke [47] and serves to highlight the need for a greater understanding of the biology underlying stress effects.

Potential mechanisms to explain the association by which psychosocial stress may increase risk of stroke are complex and not fully elucidated. Possible explanations relate to impact of perceived psychosocial stress on vascular inflammation, oxidative stress or immune dysfunction underpinning the basic pathophysiology of vascular disease [36]. Perceived stress is related to increased catecholamine release and sympathetic activation, which may either directly or indirectly affect the vascular system, eg increase thickening of the intima media, progression of carotid arterial disease and impact on blood pressure. Additionally, perceived stress adversely affects immune responses [37] which may result in increased susceptibility to complications of stroke and thus may contribute to explaining the association between perceived stress and fatal stroke in particular. It may also be the case that individuals with high perceived stress levels have more severe strokes although a mechanism for this has yet to be proposed. Additional to potential pathophysiological mechanisms, studies have also reported adverse behavioural risk profiles with regard to rates of smoking, physical activity and alcohol consumption in those who perceive themselves to be stressed [38]. The results of the meta-analysis indicating the association between perceived psychosocial stress and stroke, have potential clinical relevance suggesting interventions to manage or reduce perceived stress to be worthy of further investigation, with implications for secondary prevention of stroke. Despite the limited success of interventions to reduce psychosocial stress in primary and secondary prevention of cardiovascular disease [17], perceived psychosocial stress is theoretically modifiable. It is currently the subject of increased attention through a raised interest in interventions such as mindfulness based stress reduction [40].

### Limitations

The main limitations relate to lack of an agreed definition of perceived psychosocial stress and its measurement, therefore variation and overlap in the perceived psychosocial stress reported may have occurred. As stress was self-reported there is no objectivity in stress measures used, thus understanding and interpretation of what was asked of each participant may vary. A number of studies measured psychosocial stress using a single question, which inevitably will encompass a spectrum of individual interpretations including sensations of anxiety and depression. It is acknowledged that these constructs are difficult to separate out from perceived psychosocial stress and should be considered, although the diagnosed clinical conditions were excluded from the review. Significant heterogeneity was found across the studies which may result from differences in perceived stress measures as well as differences in study design, sample sizes, strategies for analysis and participant characteristics. Several studies examined perceived stress in a younger patient population, where the stroke incidence rate was lower than in those studies with higher proportions of older adults and this may have affected the statistical power to observe significant results. Study selection was limited to English language only, which may have resulted in missing important insights and sample sizes in some studies were small. There was variation in follow-up periods, although none shorter than 6 years and many studies focused on middle-aged men with limited women-specific data, despite the indication that impact of perceived stress may be greater for this group. It was noted that measures of perceived stress in middle age might further be confounded by other previously accumulated adversity relevant to stroke risk. One potential limitation that should be considered is that perceived stress was reported at baseline, with a follow up of 6–30 years and it cannot be assumed that the level of perceived psychosocial stress reported at baseline was consistent or sustained prior to experiencing a stroke.

## Conclusion

This meta-analysis demonstrates perceived psychosocial stress to be an independent risk factor for stroke, albeit of relatively modest magnitude. A number of hypotheses can be proposed to explain the association, however the first step is to demonstrate that a relationship exists, which this study achieves. The next steps will investigate potential moderators of the relationship, mediating mechanisms underpinning the association, and demonstration of causal links to explain the observed association.
